# Improving Bread Quality with the Application of a Newly Purified Thermostable α*-*Amylase from *Rhizopus oryzae* FSIS4

**DOI:** 10.3390/foods6010001

**Published:** 2017-01-01

**Authors:** Amel Ait Kaki El-Hadef El-Okki, Mohammed Gagaoua, Hayat Bourekoua, Kahina Hafid, Leila Bennamoun, Shahrazed Djekrif-Dakhmouche, Mohamed El-Hadef El-Okki, Zahia Meraihi

**Affiliations:** 1 Institut de la Nutrition, de l’Alimentation et des Technologies Agro-Alimentaires (INATAA), Université des Frères Mentouri-Constantine 1, Route de Ain El-Bey, 25000 Constantine, Algeria; gmber2001@yahoo.fr (M.G.); bourekoua.h@hotmail.fr (H.B.); hafidkahina@hotmail.com (K.H.); elhadef-elokki.mohamed@umc.edu.dz (M.E.E.); 2 Laboratoire de Génie Microbiologique et Applications, Université des Frères Mentouri-Constantine 1, Route de Ain El-Bey, 25000 Constantine, Algeria; leila_bennamoun@hotmail.com (L.B.); scheherazad2002@hotmail.com (S.D.-D.); meraihi27@yahoo.com (Z.M.); 3 Equipe MaQuaV, INATAA, Université des Frères Mentouri-Constantine 1, Route de Ain El-Bey, 25000 Constantine, Algeria; 4 Laboratoire de Biologie et Environnement, Université des Frères Mentouri-Constantine 1, Route de Ain El Bey, 25000 Constantine, Algeria

**Keywords:** α-amylase, *Rhizopus oryzae* FSIS4, three phase partitioning, bread quality

## Abstract

A new thermostable α-amylase from *Rhizopus oryzae* FSIS4 was purified for first time and recovered in a single step using a three-phase partitioning (TPP) system. The fungal α-amylase, at a concentration of 1.936 U per kg of flour, was used in bread-making and compared to the commercial enzyme. The results showed a significant effect of the recovered α-amylase in the prepared bread and allowed us to improve the quality of the bread. The study indicated clearly that the recovered α-amylase is a potential candidate for future applications in the bread-making industry and in other food biotechnology applications.

## 1. Introduction

Alpha-amylase (1,4-α-d-glucanglucanohydrolase, EC 3.2.1.1) is the most important carbohydrate-degrading enzyme for starch-based industries [1]. The addition of amylases in bakery products has often been reported to enhance consumer acceptance and purchase intent. Thus, amylases have been used in bread-making as standardization and anti-staling agents of flour [2]. For this purpose, fungal α-amylases were considered as very safe additives [3]. They may increase the level of fermentable sugars in the dough, thus promoting the fermentation of yeast and the formation of Maillard reaction products, which, in turn, intensify bread flavor and crust color [3]. Nevertheless, for their use, the purification of these enzymes is highly required. Purification methods of α-amylase include various traditional methods such as salting out, acid fractionation and chromatographic techniques. These methods are described to be time-consuming and involve expensive reagents and equipment. To solve these drawbacks, aqueous systems such as three-phase partitioning (TPP), known as simple, economical and quick methods, were described for the recovery of enzymes [4,5,6]. This elegant non-chromatographic tool may be performed in a purification process to be used successfully in food industries [4], namely to provide amylases for baking industries.

Considering the industrial importance of α-amylases, several works have reported on the benefits of using low-cost α-amylase processes, including the application of agro-industrial residues [7,8].

In this context, the present investigation was aimed at studying the α-amylase production by *Rhizopus oryzae* FSIS4 in a laboratory fermentor using an agro-industrial residue (decommissioned date) as the base medium. The performance of the enzyme preparation from *R. oryzae* FSIS4 was tested in the bread-making process and compared to a currently commercialized amylase

## 2. Materials and Methods

### 2.1. Microorganism and Inoculum Preparation

The fungal strain *R. oryzae* FSIS4 (GenBank Accession No. KU726976.1) isolated from the wheat seed cultivated in an arid area from Algerian Sahara [9] was propagated on Potato Dextrose Agar (PDA) medium plate at 40 °C. The spores were harvested from a seven-day-old culture by dislodging them in sterile water. The obtained suspension was used as inoculums after adjustment to the desired concentration (10^6^ spores/mL). A Thoma cell was used for counting.

### 2.2. Medium Composition and Fermentation

The culture medium contained (flour decommissioned dates 200 g/L, starch 5.42 g/L, yeast extract 2.3 g/L, CaCl_2_ 0.47 g/L and MgSO_4_ 0.39 g/L. The contents were thoroughly mixed, and the initial pH was adjusted to 5.0 [9].

Batch fermentations were performed in a 5 L BIOSTAT^®^ Aplus (Sartorius Stedim Biotech GmbH, Goettingen, Germany) with a working volume of 3 L. The system was interfaced with the BioPAT^®^ MFCS/win software (version 3, Sartorius Stedim Biotech GmbH, Goettingen, Germany) to register data and control the external peripherals. Fermentations were inoculated with spores at a level of 10^6^ spores/mL. Throughout the batch process, a fermentation temperature of 50 °C, a pH value of 5.0, an aeration rate of 1.0 vvm and an agitation rate of 100 rpm were kept constant.

### 2.3. Partial Purification and Enzyme Formulation

The crude extract was collected after 48 h of batch incubation. After a centrifugation at 4000 rpm for 15 min, TPP experiments were carried out as described by Dennison and Lovrien [10] following the recommendations of Gagaoua et al. [11]. The enzyme exclusively recovered in the interfacial phase was gently separated from the other phases and dissolved in 30 mM, pH 6.5 phosphate buffer and dialyzed overnight against distilled water at 5 °C. The enzyme was migrated in 12% SDS PAGE in accordance with the method of Laemmli [12]. Protein bands were visualized by Coomassie brilliant blue R-250 (Bio-Rad, Strasbourg, France) staining.

A volume of purified extract enzyme was mixed with three volumes of dehydrated starch, and the mixture was then dried at 42 °C for 48 h, yielding a white powder of enzyme preparation [13].

### 2.4. Amylase Activity and Protein Content

Alpha-amylase was assayed by the addition of 0.5 mL of the culture supernatant to 0.5 mL of 1% (*w*/*v*) starch, which was dispersed in 0.1 M phosphate buffer (pH 5). The reaction mixture was incubated for 30 min at 40 °C, and the liberated reducing sugars were measured using the 3,5-dinitrosalicylic acid method [14]. A separate blank was made for each sample to eliminate the non-enzymatic release of sugars. One unit of the α-amylase activity corresponded to the amount of enzyme that released reducing sugars equivalent to 1 µmoLmaltose per min under the standard assay conditions.

Protein concentration was determined according to Bradford [15] using bovine serum albumin as a standard.

### 2.5. Determination of Carbohydrate Content and Analysis of Hydrolysis Products

The purified amylase was incubated with 1% starch in 100 mM sodium phosphate buffer (pH 5.0) at 60 °C. Samples were removed after 0.5, 1, 2, 3 and 24 h incubation, and hydrolysis was stopped by heating the samples in boiling water for 3 min. The products were detected by thin-layer chromatography (DC-Alufolien Kieselgel 60, Merck, Darmstadt, Germany), as described by Fontana et al. [16].

### 2.6. Bread-Making

The performance of the enzyme preparation from *R. oryzae* FSIS4 was tested in the bread making process using wheat flour (from a local milling industry) with a relative humidity of 14.66% ± 0.41%. The results were compared to a currently commercialized enzyme (Fungamyl^®^ α-amylase from Novozymes, Bagsværd, Denmark) from *Aspergillus oryzae*. Bread was prepared according to Ndangui et al. [17]. Dough consisted of wheat flour (100 g), salt (2 g), dry baker’s yeast (2 g), 65 g of water and α-amylase which consisted of 1.936 U per kg of flour, corresponded to the standard commercial amylase concentration [13].

A separate control was set up to test the non enzymatic release of sugars with the same protocol and without the supplementation of α-amylase.

All the ingredients were mixed for 20 min. Dough was left to rest for 45 min at 35 °C with 75% of relative humidity. The resulting dough was divided in lumps (70 g) and put into mold and proofed for 35 min at 35 °C in a fermentation cabinet. The baking tests were carried out at 210 °C for 20 min into an electric oven.

### 2.7. Bread Quality Evaluation

Bread characterization after 1 h post-baking consisted of specific volume and height/width ratio.

Loaf volume was determined by a colza seed displacement method wherein the loaf was placed into a container of a known volume filled by small colza seeds. The volume of seed displaced by the bread loaf was directly indicated. Analyses were performed in duplicate according to the AACC Approved Method 10.05 [18].

Specific volume (cm^3^/g) of an individual loaf was calculated by dividing volume by weight. Height/width ratio was measured by capturing the image of the central slice with an HP Scanjet G 3110 scanner in the presence of scale.

### 2.8. Statistical Analysis

Analysis of variance (ANOVA) was applied to compare the effect of addition of *R. oryzae* FSIS4 α-amylase and currently commercialized α-amylase on specific volume and height/width ratio with R software (R Foundation, Vienna, Austria) [19].

## 3. Results and Discussion

### 3.1. Purification of α-Amylase

**Figure 1:**
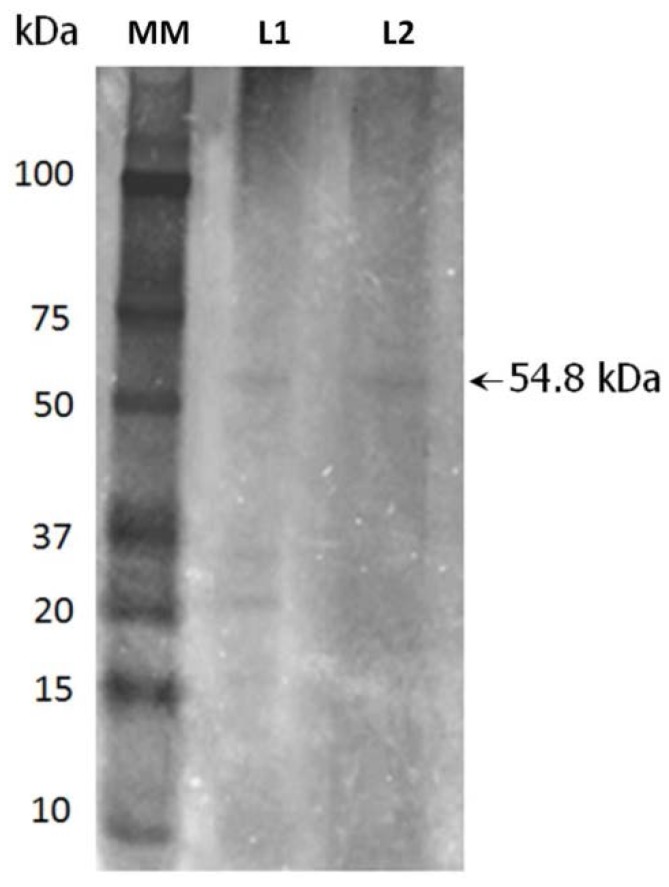
SDS-PAGE profile of α-amylase purified from *R. oryzae* FSIS4: Lane MM, molecular marker; Lane L1, dialyzed crude extract; Lane L2, purified α-amylase (interfacial phase) with an apparent molecular mass of 54.8 kDa.

The α-amylase from the crude extract of *R. oryzae* FSIS4 was efficiently recovered using the TPP technique. A purification fold of 14.94 and a recovery yield of 168.8% were obtained. Using macroaffinity ligand-facilitated TPP, Mondal et al. [20] purified three α-amylases from wheat germ (55-fold), *Bacillus amyloliquefaciens* (5.5-fold) and porcine pancreas (10-fold) with recovery yields of 77%, 74%, and 92%, respectively. Recently, Sagu et al. [21] purified β-amylase from stems of *Abrus precatorius* using a single-step TPP. The authors found an activity recovery of 156.2% and a purification factor of 10.17. Similarly, several studies reported high recovery yields (>100%) for the purification of proteases using the TPP system [4]. On the other hand, Tricine SDS-PAGE analysis showed that the recovered α-amylase in this study showed a single band with an apparent molecular weight of 54.8 kDa (Figure 1).

### 3.2. TLC of Hydrolysis Products

**Figure 2:**
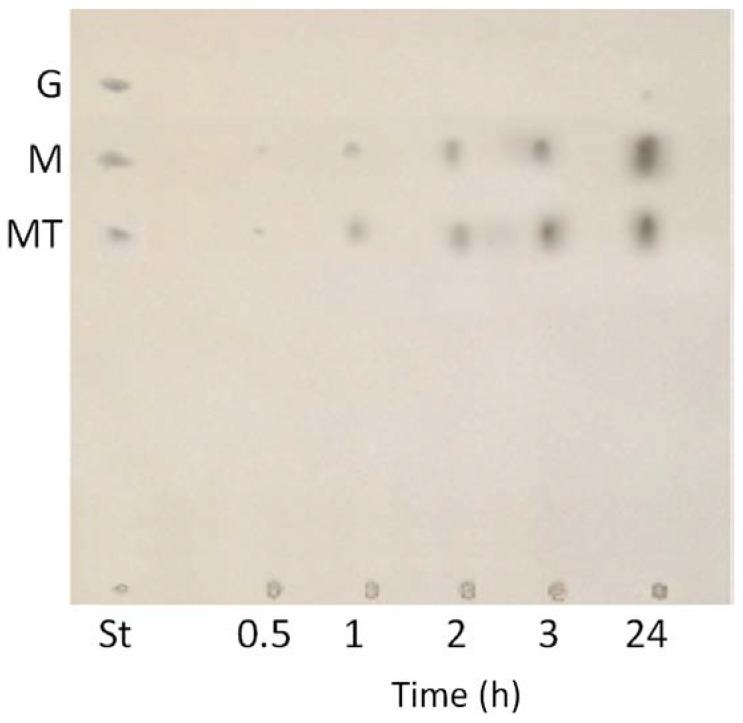
Thin-layer chromatography of the reaction products of soluble starch hydrolyzed by the purified α-amylase. Hydrolysis times were 0.5, 1, 2, 3 and 24 h. Standards (St) were a mixture of 1 mg/mL of glucose (G), maltose (M), maltotriose (MT).

The classification of the *R. oryzae* FSIS4 enzyme as α-amylase or glucoamylase was based mainly on the products of starch hydrolysis. After a reaction period of 0.5–24 h, the α-amylase products formed against starch were mainly maltose and maltotrioses (Figure 2). These results indicated the endo-amylolytic character of the enzyme, which was classified as an α-amylase.

### 3.3. Bread-Making Performances

**Figure 3:**
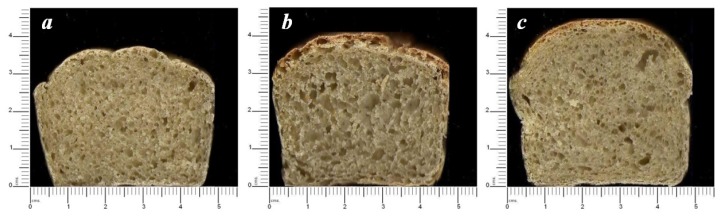
Cross-section of central bread slice: (**a**) control; (**b**) with commercial α-amylase; (**c**) with *R. oryzae* FSIS4 α-amylase.

The results obtained from the baking tests performed for the formulated, commercial, and control α-amylases are summarized in Figure 3.

The findings revealed that the application of the formulated α-amylase gave satisfactory crust bread colors (Figure 3c) which were nearly similar to those obtained with the commercial enzyme used by local manufacturers (Figure 3b). The image crumb analysis revealed that bread obtained with formulated α-amylase contained a higher number of small holes, whereas bread obtained with commercial α-amylase contained bigger holes. In both cases, breads exhibited an aerated structure (Figure 3b,c), which is likely to be the result of an optimum production of carbon dioxide in the dough for a uniform honey comb-like texture [22]. The pale or grayish crust color obtained with the control indicated a lack of residual sugars which might have resulted from a lean fermentation (Figure 3a).

The addition of the formulated α-amylase was noted to induce an increase in the specific volume and height/width ratio of the bread of 0.72 and 0.2, respectively (Table 1), as compared to the control. This increase in the bread volume and height/width ratio was higher than the one induced by the commercial α-amylase, which attained 0.49 and 0.1, respectively (highly significant difference with *F* = 33.85 (*p* < 0.001) for the specific volume and *F* = 74.72 (*p* < 0.001) for the height/width ratio). It is presumably related to the reduction of the dough viscosity during starch gelatinization as a result of the action of the enzyme [23].

In order to achieve a good bread volume, the dough should have enough strength to develop and maintain the cells’ gas and gelatinized starch must be able to withstand the rapid expansion of cells during the initial phase of the cooking [24].

## 4. Conclusions

Rapid advances in biotechnology have made a number of exciting new enzymes available for the baking industry. This report showed clearly that the recovered α-amylase from *R. oryzae* FSIS4, using the fast and efficient emerging TPP technique, represents a potential biotechnological enzyme. The formulated fungal α-amylase, at a concentration of 1.936 U per kg of flour, gave better results in terms of the bread specific volume and height/width ratio. The addition of the formulated *R. oryzae* FSIS4 α-amylase may be a potentially strong candidate for future applications in the bread-making industry.

## Figures and Tables

**Table 1:** Evaluation of α-amylase activity on specific volume and height/width ratio.

	Specific Volume (cm^3^/g)	Height/Width (Ratio)
Without *α-amylase*	1.99 ± 0.21 ^a^	0.79 ± 0.01 ^a^
With commercial *α-amylase*	2.48 ± 0.08 ^b^	0.89 ± 0.02 ^b^
With *α-amylase* of *R. oryzae* FSIS4	2.71 ± 0.14 ^c^	0.98 ± 0.02 ^c^

^a,b,c^: different letters indicate statisticallysignificant differences between tested *α-amylases* at *p* < 0.05 by Student-Newman-Keuls post-hoc test.
